# Society Meetings

**Published:** 1899-12

**Authors:** 


### SOCIETY MEETINGS.

The Esculapian Club held its second regular meeting at the residence of Dr. C. J. Carr, November 16, 1899. Dr. Charles E. Congdon read a paper on Cancer of cervix uteri, with a report of twelve cases. The next meeting will be held at Dr. F. J. Carr's, December 21, 1899, when Dr. J. W. Charters will read a paper.

---

The Buffalo Academy of Medicine held meetings during the month of November, 1899, as follows:

*Section on Surgery.*—Tuesday evening, November 7th. Program: Etiology and pathology of appendicitis and their bearing on the treatment, by Herman Mynter.

*Section on Medicine.*—Tuesday evening, November 14th. Program: Protracted pneumonia and slow resolution, by John H. Pryor; Hygiene of the asthmatic, by George N. Jack, Depew, N. Y.

*Section on Pathology.*—Tuesday evening, November 21. Program: The relation of dentition to disease, by C. S. Butler, Esq., D.D.S.; discussion opened by Irving M. Snow.

*Section on Obstetrics.*—Tuesday evening, November 28th. Lessons from practice in obstetrics, by P. W. Van Peyma; Mensuration and capacity of the female bladder, Irving P. Lyon.

---

The Niagara Falls Academy of Medicine held its last regular meeting at the office of Dr. W. A. Scott, 610 Pine Avenue, Monday, November 6, 1899, at 8.45 p. m. A paper was read by Dr. Scott and several matters of importance were discussed at this meeting.

